# Epidemiology of anorexia nervosa in Japanese adolescents

**DOI:** 10.1186/s13030-015-0044-2

**Published:** 2015-08-14

**Authors:** Mari Hotta, Reiko Horikawa, Hiroyo Mabe, Shin Yokoyama, Eiko Sugiyama, Tadato Yonekawa, Masamitsu Nakazato, Yuri Okamoto, Chisato Ohara, Yoshihiro Ogawa

**Affiliations:** Health Services Center, National Graduate Institute for Policy Studies, 7-22-1 Roppongi, Minato-ku, Tokyo, 106-8677 Japan; Division of Endocrinology and Metabolism, National Center for Child Health and Development, 2-10-1 Okura, Setagaya-ku, Tokyo, 157-8535 Japan; Department of Child Development, Kumamoto University Hospital, 1-1-1 Honjo, Chuo-ku, Kumamoto, 860-8556 Japan; Department of Psychiatry, Nagano Red Cross Hospital, 5-22-1 Wakasato, Nagano, 380-8582 Japan; Department of Health and Nutrition, Nagano Prefectural College, 8-49-7 Miwa, Nagano, 380-8525 Japan; Division of Neurology, Respiratory, Endocrinology and Metabolism, Department of Internal Medicine, Faculty of Medicine, University of Miyazaki, 5200 Kihara, Kiyotake-machi, Miyazaki, 889-1692 Japan; Health Service Center, Hiroshima University, 1-2-3 Kasumi, Minami-ku, Hiroshima, 734-8551 Japan; Institute of Women’s Health, Tokyo Women’s Medical University, 9-9 Wakamatsu-cho, Shinjuku-ku, Tokyo, 162-0056 Japan; Department of Molecular Endocrinology and Metabolism, Graduate School of Medical and Dental Sciences, Tokyo Medical and Dental University, 1-5-45 Yushima, Bunkyo-ku, Tokyo, 113-8519 Japan

**Keywords:** Anorexia nervosa, Epidemiology, Prevalence, Adolescent, Eating disorder center, Japan

## Abstract

**Background:**

No epidemiologic survey examining eating disorders in Japan has been done at a national level since 1992. The prevalence of anorexia nervosa, as assessed by questionnaires to hospitals, is thought to be underestimated because patients with anorexia nervosa tend to avoid consultations. In conformity with the School Health and Safety Act of Japan, schools are required to have physicians perform a medical examination of students every year. The teachers in charge of health education and school physicians determine the height, weight, and health condition, and examine the medical records of each student. Therefore, we as members of the Survey Committee for Eating Disorders of the Japanese Ministry of Health, Labour, and Welfare conducted an epidemiologic survey using questionnaires sent to schools in seven prefectures to determine the current prevalence of anorexia nervosa among adolescents.

**Methods:**

We sent a questionnaire to elementary, junior high, and senior high schools. Questionnaires contained items on the number of students, patients with anorexia nervosa in each grade who were diagnosed by specialists, and students who the school physician strongly suspected to have anorexia nervosa but who did not undergo a clinical examination in a medical institution.

**Results:**

We found patients of both sexes with anorexia nervosa aged 9–10 years in elementary schools. The point prevalence of anorexia nervosa for girls, including strongly suspected cases, in the three grades of junior high school and three grades of senior high school were 0–0.17 %, 0–0.21 %, 0.17-0.40 %, 0.05-0.56 %, 0.17-0.42 % and 0.09-0.43 %, respectively. We also confirmed a prominent sex difference in the prevalence of anorexia nervosa. The prevalence of boys was one third that of girls in some prefectures. One third to one half of diagnosed and strongly suspected students with anorexia nervosa had not received medical consultation or treatment.

**Conclusions:**

Although the prevalence of anorexia nervosa had regional differences in Japan, it has reached levels comparable to those in Western societies. Because no eating disorder center exists and the treatment environment is poor, national action to address this disease is a pressing need in Japan.

## Background

Anorexia nervosa is associated with various types of medical morbidity due to malnutrition [1] and a significant mortality rate in adolescents [2]. The last survey performed in Japan at a national level was done in 1992 by the Survey Committee for Eating Disorders of the Japanese Ministry of Health, Labour and Welfare who sent questionnaires to hospitals with 300 or more beds [3]. The prevalence of anorexia nervosa of female patients aged 10 to 29 years was 0.025 % to 0.031 %. Based on a questionnaire sent to hospitals and clinics in Niigata prefecture, which faces the Sea of Japan and had the 14^th^ largest population in Japan in 1997, the prevalence of anorexia nervosa of female residents aged 15 to 29 years was 0.017 % [4]. In contrast, the prevalence of anorexia nervosa as assessed by questionnaires returned by schools was higher than that obtained from medical facilities [3]; in that study, the prevalences of anorexia nervosa for the junior and senior high school girls of Kyoto were 0.24 % and 0.15 %, respectively. It was thought that the prevalence obtained based on questionnaires sent to medical facilities might underestimate disease prevalence because patients with anorexia nervosa may refuse or not seek consultation [5].

In conformity with the School Health and Safety Act of Japan, school physicians have to perform a medical examination for students every year. The teachers in charge of health education in Japan complete a curriculum for adolescent health and some have a school-nurse license. Every school has a school physician, whose specialty is internal medicine, pediatrics, or gynecology. The teachers in charge of health education and school physicians determine height, weight, and health condition and review the medical records of each student.

School physicians and teachers in charge of health education list students who show poor weight gain, lose weight in comparison with the last year, or have a body weight lower than 80 % of the expected body weight in this annual medical examination. The expected weight of a child is based on height and indexes prescribed to age and gender. Growth curves are also drawn to identify growth retardation. These health care professionals usually see students with very low weight to identify the clinical characteristics of anorexia nervosa, including amenorrhea, lanugo, bradycardia, constipation, hypotension, hypothermia, and edema, which are described in the diagnostic criteria of The Survey Committee for Eating Disorders of the Japanese Ministry of Health, Labour and Welfare, and also ask the students questions according to the Diagnostic and Statistical Manual fourth edition (DSM-IV) criteria. When a student is a strongly suspected case of anorexia nervosa, the school physician advises the parents or guardians to consult with a specialist.

In this study, the Survey Committee performed an epidemiologic survey of anorexia nervosa by sending questionnaires to schools in seven prefectures in Japan to obtain updated prevalence data.

## Methods

A questionnaire was sent to principals and the teachers in charge of health education at junior and senior high schools in Tokyo, Hokkaido, Hiroshima, Nagano, Kumamoto, Yamaguchi, and Miyazaki, which had respective population rankings during this survey of 1, 8, 12, 16, 23, 25, and 36 of the 47 Japanese prefectures, and to elementary schools in six prefectures (same as the above except for Hokkaido). Tokyo is the capital of Japan. Hokkaido is the northernmost prefecture. Nagano is a mountainous prefecture in the center of the country. Hiroshima and Yamaguchi are located in the west of Honshu, the main island. Kumamoto and Miyazaki prefectures are located on the island of Kyushu. The questionnaire gathered background on each school, and the number of students with anorexia nervosa, including those with strongly suspected anorexia nervosa, in each grade at the time of the survey. We used the DSM-IV criteria for anorexia nervosa in addition to the criteria for anorexia nervosa put forth by the Survey Committee for Eating Disorders of the Japanese Ministry of Health, Labour, and Welfare (Table 1) [6]. The build of Japanese is smaller than that of Caucasian. Based on the DSM-IV criteria, many healthy girls with regular menstruation who are at 85 % of normal weight are selected for screening for anorexia nervosa. In this study, we used a body weight less than 80 % of the ideal body weight based on the Japanese criteria to identify patients with anorexia nervosa. In the present study, we may have overlooked patients with anorexia nervosa who met only the DSM-IV criteria.

**Table 1:** The diagnostic criteria of anorexia nervosa put forth by The Survey Committee for Eating Disorders of Japanese Ministry of Health, Labour and Welfare

1	Weight loss of more than 20 % of normal weight, lasting longer than 3 months.
2	Abnormal eating behavior involving food restriction, bulimic episodes, and eating by stealth.
3	Disturbances in the way in which one’s body weight or shape is experienced and intense fear of gaining weight, even though underweight.
4	Onset younger than 30 years.
5	In females, amenorrhea, as well as other clinical symptoms including lanugo, bradycardia, constipation, hypotension, hypothermia or edema.
6	Negation of illness including other psychiatric disorders that account for anorexia and weight loss.

The questionnaire did not ask the teachers in charge of health education or the school physicians about the types of anorexia nervosa because they may not always recognize the binge eating/purging-type without a report from the student.

Japanese compulsory education begins when children are six to seven years old. They study for six years at an elementary school and then three years at a junior high school. The percent of junior high school students who go on to senior high school in Japan is more than 97 %. Japanese senior high schools use a three-year system. A senior high school student’s age ranges from 15 to 18 years. We provided a written explanation of this study to the parents of the students in each school. Based on the judgment of the principal, personal data can be provided to a research team when its goal is to benefit students and the data are provided anonymously. The study protocol was approved by the institutional review board of each author. This study was also carried out with the permission of the Board of Education in each of the seven prefectures.

## Results and discussion

Table 2 shows data on the total number of students enrolled in the seven prefectures according to the School Basic Survey by the Japanese Ministry of Health, Labour and Welfare, the number of subjects who participated in this study, the participation rate, and the number of students diagnosed with anorexia nervosa, including those with strongly suspected anorexia nervosa, for each grade. Participation rates for the questionnaire varied by school and by prefecture. In Tokyo, elementary and junior high schools in a limited area participated in the study and those subjects represented only about 5 % of the total number of children. Although the Education Bureau of Tokyo prefecture requested schools to cooperate with our survey and we sent a collection letter to schools that did not provide answers, some schools seemed to resist information disclosure. In contrast, about 60 % of students in every grade in Yamaguchi and Kumamoto participated in the research.

**Table 2:** Prevalence of anorexia nervosa (AN) in adolescents from 7 prefectures in Japan

Prefecture year of survey	Sex	Data	Elementary school grade				Junior high school year			Senior high school year		
			3th	4th	5th	6th	1st	2nd	3rd	1st	2nd	3rd
Tokyo 2010	Girls	Total			48652	48886	51178	51657	50766	53332	50046	48390
		Subjects			2893	2900	2256	2285	2259	22205	22329	21550
		% Total			5.9	5.9	4.4	4.4	4.4	41.6	44.6	44.5
		No. with AN			2	3	2	4	9	47	60	56
		Prevalence of AN			0.07	0.10	0.09	0.18	0.40	0.21	0.27	0.26
	Boys	Total			51049	51230	51833	52367	51446	51091	47990	46466
		Subjects			2893	2834	2166	2149	2092	15228	15140	13828
		% Total			5.7	5.5	4.2	4.1	4.1	29.8	31.5	29.8
		No. with AN			0	1	0	1	1	2	1	2
		Prevalence of AN			0.00	0.04	0.00	0.05	0.05	0.01	0.01	0.01
Kumamoto 2011	Girls	Total			8432	8490	8498	8603	8804	8379	8390	8062
		Subjects			4860	4997	5113	5116	5150	5206	5131	5044
		% Total			57.6	58.9	60.2	59.5	58.5	62.1	61.2	62.6
		No. with AN			0	1	2	5	10	9	12	18
		Prevalence of AN			0.00	0.02	0.04	0.10	0.19	0.17	0.23	0.36
	Boys	Total			9052	8880	9134	9123	9394	8823	8727	8280
		Subjects			5027	5207	5277	5490	5451	6116	5784	5643
		% Total			55.5	58.6	57.8	60.2	58.0	69.3	66.3	68.1
		No. with AN			0	0	0	0	0	2	2	2
		Prevalence of AN			0.00	0.00	0.00	0.00	0.00	0.03	0.04	0.04
Nagano Seniors 2011 others 2012	Girls	Total			10074	10236	10298	10304	10346	10056	10211	9274
		Subjects			8746	8629	9120	9165	9195	2589	2644	2396
		% Total			87.0	84.3	88.6	88.9	88.9	25.7	25.9	25.8
		No. with AN			6	9	11	14	22	4	7	4
		Prevalence of AN			0.07	0.10	0.12	0.15	0.24	0.15	0.27	0.17
	Boys	Total			10659	10875	10926	10858	10939	10255	10436	9678
		Subjects			9222	9166	9631	9658	9600	2239	2405	2279
		% Total			86.5	84.3	88.1	88.9	87.8	21.8	23.0	23.5
		No. with AN			2	2	0	2	2	0	0	0
		Prevalence of AN			0.02	0.02	0.00	0.02	0.02	0.00	0.00	0.00
Hiroshima 2013	Girls	Total		12740	12728	13155	12953	13050	13277	12655	12360	11870
		Subjects		4454	4383	4426	4122	4213	4736	2849	2808	2798
		% Total		35.0	34.4	33.6	31.8	32.3	35.7	22.5	22.7	23.6
		No. with AN		4	3	6	7	9	16	16	12	12
		Prevalence of AN		0.09	0.07	0.14	0.17	0.21	0.34	0.56	0.43	0.43
	Boys	Total		13323	13438	13785	13699	13693	14025	12824	12436	11838
		Subjects		4561	4646	4426	4411	4641	4399	2290	2242	2202
		% Total		34.2	34.6	32.1	32.2	33.9	31.4	17.9	18.0	18.6
		No. with AN		1	1	1	0	1	1	0	1	0
		Prevalence of AN		0.02	0.02	0.02	0.00	0.02	0.02	0.00	0.05	0.00
Miyazaki Seniors 2013 others 2012	Girls	Total		5201	5333	5445	5321	5378	5700	5433	5283	5197
		Subjects		1008	1074	1082	333	337	336	4187	4173	4270
		% Total		19.4	20.1	19.9	6.3	6.3	5.9	77.1	79.0	82.2
		No. with AN		0	0	0	0	0	1	2	7	4
		Prevalence of AN		0.00	0.00	0.00	0.00	0.00	0.30	0.05	0.17	0.09
	Boys	Total		5399	5655	5594	5568	5882	5873	5600	5540	5327
		Subjects		1024	1141	1066	341	378	343	4488	4410	4250
		% Total		19.0	20.2	19.1	6.1	6.4	5.8	80.1	79.6	79.8
		No. with AN		0	0	0	0	0	0	0	1	0
		Prevalence of AN		0.00	0.00	0.00	0.00	0.00	0.00	0.00	0.02	0.00
Yamaguchi 2013	Girls	Total	5751	5943	6154	6346	6198	6228	6354	5863	5820	5594
		Subjects	3215	3237	3433	3615	3567	3578	3676	3930	3897	3714
		% Total	55.9	54.5	55.8	57.0	57.6	57.5	57.9	67.0	67.0	66.4
		No. with AN	0	1	3	2	5	1	7	5	11	10
		Prevalence of AN	0.00	0.03	0.09	0.06	0.14	0.03	0.19	0.13	0.28	0.26
	Boys	Total	6126	6187	6266	6665	6370	6606	6621	5947	5773	5596
		Subjects	3367	3440	3468	3718	3645	3786	3740	3990	3866	3734
		% Total	55.0	55.6	55.3	55.8	57.2	57.3	56.5	67.1	67.0	66.7
		No. with AN	0	1	0	0	1	1	0	2	3	1
		Prevalence of AN	0.00	0.03	0.00	0.00	0.03	0.03	0.00	0.05	0.08	0.03
Hokkaido 2013	Girls	Total					22345	22500	23247	22055	21579	21424
		Subjects					8924	9007	9211	10170	9667	9582
		% Total					39.9	40.0	39.6	46.1	44.8	44.7
		No. with AN					7	8	16	6	18	18
		Prevalence of AN					0.08	0.09	0.17	0.06	0.19	0.19
	Boys	Total					23096	23443	24326	21130	22154	21256
		Subjects					9107	9217	9608	9569	9592	9230
		% Total					39.4	39.3	39.5	45.3	43.3	43.4
		No. with AN					1	2	4	1	1	2
		Prevalence of AN					0.01	0.02	0.04	0.01	0.01	0.02

Total: a number of all children or students in a prefecture according to the School Basic Survey by the Japanese Ministry of Health, Labour and Welfare

Subjects: a number of subjects in the present study

We confirmed that anorexia nervosa was predominant in girls. This is the first study in Japan that examined the prevalence of anorexia nervosa among children 12 years old or younger. We found patients of both sexes in the fourth grade in elementary schools (aged 9–10 years) with anorexia nervosa. For junior high school girls, the prevalence increased in a straight line and became highest among third-year students. For senior high school girls, the peak prevalence was observed in the second or third year in all prefectures except for Hiroshima. The point prevalences of anorexia nervosa for girls, including strongly suspected cases in the three grades of junior high school and three grades of senior high school were 0–0.17 %, 0–0.21 %, 0.17-0.40 %, 0.05-0.56 %, 0.17-0.42 %, and 0.09-0.43 %, respectively. We also confirmed a prominent sex difference in the prevalence of anorexia nervosa, with the prevalence of boys one third that of girls in senior high schools in Yamaguchi. One third to one half of those diagnosed with anorexia nervosa or with strongly suspected anorexia did not receive medical treatment (data not shown).

We demonstrated the recent prevalence of anorexia nervosa, including strongly suspected cases of anorexia nervosa, among adolescents in seven prefectures in Japan. We cannot show evidence to explain the regional differences in the prevalence at the present stage. Although there were regional differences among prefectures, the prevalence of anorexia nervosa in this study was similar or higher than the rates reported in a previous epidemiologic study using the same survey method as the present study [3]. Our report is also the first on the prevalence of anorexia nervosa among elementary school children. Because anorexia nervosa before menarche can retard growth and lower peak bone mass [7, 8] preventing this disorder, early detection, early consultation, and communication between parents, physicians, and schools are pressing needs.

Since the 1960s, eating disorders such as anorexia nervosa began to be recognized as an important health problem among adolescent girls and young women in Western societies, and their prevalence has increased over time [9]. Although the survey methods and ages of the subjects in other studies differed from those in our study, the prevalence of anorexia nervosa was shown to be 0.3 % of young females in the Netherlands [10], 0.5 % of teenaged girls in Germany [11] 0.3 % of secondary school students in Hungary [12], and 0.2-0.3 % of girls aged 13 to 18 years in the United States [13]. Although it was previously reported that the prevalence of anorexia nervosa in Japan is lower than that for European Caucasian populations [4], we showed that the prevalence among adolescents in Japan is near that of developed European countries and the United States.

As shown in other studies [14, 15], adolescent girls with eating disorders often refuse consultations with a physician, never seek treatment, or are untreated. One third to one half of the students with anorexia nervosa in the present study had not consulted physicians. Unfortunately, there is no eating disorder center in Japan, which leads to a poor treatment environment. The establishment of an eating disorder center can contribute at a national level to education for physicians and paramedical professionals, research support, and a public awareness campaign on eating disorders. Medical administration policies should be considered based on the results of the current study. Few hospitals employ specialists in eating disorders. These two factors may contribute to the low consultation rates found in the present study.

There are several critical limitations to this study. First, prevalence was estimated using indirect information obtained from schools. It is possible that teachers in charge of health education and school physicians were unable to identify milder cases of anorexia nervosa. Second, only seven prefectures participated. Many prefectural Boards of Education refused to participate because of the pressure of additional work, and some schools refused to participate due to privacy concerns. Third, participation rates for the questionnaire varied by school and by prefecture.

## Conclusions

In conclusion, medical facilities and treatment environments conductive to the treatment of for eating disorders need to be developed in Japan because the prevalence of anorexia nervosa among adolescents in this survey was similar to that found in European countries and the United States. It is clear that this health concern needs to be addressed.
